# A Comparative Analysis of Milk Oligosaccharides via LC-MS: Globally Distributed Cattle Breeds and Native Northern Finncattle

**DOI:** 10.3390/biology13110855

**Published:** 2024-10-23

**Authors:** Yu Wang, Yu-Ping Huang, Mana Rogers, Heidi Leskinen, Päivi Soppela, Anne Tuomivaara, Juha Hyvönen, Daniela Barile

**Affiliations:** 1 Food Science and Technology, University of California, 595 Hilgard Ln, Davis, CA 95616, USA; 2 Natural Resources Institute Finland, Tietotie 4, 31600 Jokioinen, Finland; 3 Arctic Centre, University of Lapland, Pohjoisranta 4, 96200 Rovaniemi, Finland; 4 Natural Resources Institute Finland, Ounasjoentie 6, 96200 Rovaniemi, Finland

**Keywords:** dairy, cattle breed, native breed, genetic diversity, milk, oligosaccharides, liquid chromatography–mass spectrometry

## Abstract

Milk oligosaccharides are complex carbohydrates composed of various monosaccharide units linked together by glycosidic bonds. They play an essential role in promoting gut health by fostering beneficial bacteria, supporting the development of the immune system, and protecting against infections and diseases. This work compared the oligosaccharide profiles in widely utilized breeds such as Holstein and Ayrshire (Nordic Red), with the native Northern Finncattle, which is considered an endangered breed. Oligosaccharides were extracted from milk and analyzed by liquid chromatography–mass spectrometry. The composition and relative abundance of the identified oligosaccharides were characterized and compared. The statistical analyses showed that neutral, sialylated, and fucosylated oligosaccharides vary among the breeds. Ayrshire and Northern Finncattle oligosaccharides formed a cluster, while Holstein’s profile shared features with both Ayrshire and Northern Finncattle. Holstein had the lowest abundance of fucosylated OS among the three breeds, with Ayrshire having the highest content followed by Northern Finncattle. The relatively higher sialylated over neutral content of Northern Finncattle is an important feature that should be preserved. Ayrshire is a good candidate to recover more diverse oligosaccharides with potential gut health implications for consumers.

## Simple Summary

This study is the first to elucidate the milk oligosaccharide profile of globally distributed cattle breeds such as Holstein and Ayrshire, which are widely utilized in the dairy industry, with the endangered Northern Finncattle breed, native to Lapland, Finland. Oligosaccharides are sugars with complicated structures that play crucial roles in neonatal development and gut health. We investigated the variations in neutral, sialylated, and fucosylated oligosaccharides among these breeds. Although similar oligosaccharide distributions were observed in all breeds, Ayrshire milk was shown to contain oligosaccharides with more intricate structures, whereas Northern Finncattle and Holstein milk contained a higher level of sialylated oligosaccharides, which are known for their role in promoting brain development. Holstein had the lowest abundance of fucosylated oligosaccharides among the three breeds. These findings enhance our understanding of the milk composition of cattle breeds, which can inform breeding programs and dairy product development, ultimately contributing to improved nutritional and health outcomes in society. In addition, these findings indicate significantly different OS content in NF, which may inspire further preservation efforts based on enhancing genetic diversity.

## 1. Introduction

Across the mammalian kingdom, milk is a vital first food for mammalian newborns. Milk is a nutrient-dense source and supports rapid growth and development. It provides essential nutrients, including proteins, lipids, carbohydrates, vitamins, and minerals [1,2]. Additionally, milk contains oligosaccharides (OS), a class of indigestible carbohydrates comprising three–fifteen monosaccharide units connected by a variety of glycosidic linkages. In human milk, over 200 OS structures have been identified [3], with a total concentration of 5–20 g/L, making OS the third most abundant solid component [4,5] after lactose and lipids. OS have been associated with multiple health and nutrition outcomes. These indigestible sugars act as prebiotics, feeding beneficial gut bacteria [6,7] and supporting immune functions [8,9,10]. Milk-derived OS have also been shown to directly prevent harmful bacteria from attaching to the gut lining [11,12], reducing the risk of infections [13,14], a function that is mostly carried out by the building blocks fucose and sialic acid. Additionally, some OS, containing those same building blocks, show promise in promoting brain development [15,16], making milk a true powerhouse for a healthy start to life. Bovine milk OS share many of the same building blocks and structural features as those in human milk [17,18]. The monosaccharide building blocks found in bovine milk include glucose (Glc), galactose (Gal), *N*-acetylglucosamine (GlcNAc), *N*-acetylgalactosamine (GalNAc), fucose (Fuc), *N*-acetylneuraminic acid (NeuAc), and *N*-glycolylneuraminic acid (NeuGc). There is growing evidence showing that bovine milk-derived OS showcase similar health benefits to those found in human milk [19,20]. Investigating the OS from bovine milk enhances our comprehension of their roles in mammalian neonatal development. Moreover, recognizing the bioactive properties of OS contributes to the advancement of functional foods and the development of innovative dairy products.

The synthesis of OS is not strictly controlled by templates like in the case of DNAs and proteins. A known factor that can influence the production of bovine milk oligosaccharides is the lactation period [21,22] as the concentration and composition of OS in bovine milk undergo significant changes throughout lactation. Colostrum, the first secretion produced after birth, has the highest concentration of OS and other immune factors and has been shown to be vital for newborn calves. Then, the OS concentration rapidly declines during the transition to mature milk, due to the changing nutritional and immunological needs of the newborn. There are also data showing that grazing management can affect the concentration of one of the OS building blocks, sialic acid [23]. Diet can also play a role, to some extent. Feeding a high-fiber, low-starch diet resulted in a more positive percent change for some OS (including the less abundant fucosylated ones) than a high-starch, low-fiber diet on milk from the same set of Holstein cows in a crossover study [24]. Other studies have shown that the cow’s parity had a significant impact on the OS profile [24,25], although the conclusions on parity were inconsistent. Cows of different breeds have also been shown to produce structurally diverse OS. For example, Jersey and Holstein cattle [25,26], along with native European breeds [27], exhibit distinct variations in OS composition, reflecting their different genetic backgrounds and adaptations.

The Northern Finncattle is a heritage breed from northern Finland. This breed is well adapted to the cold climate and harsh conditions near the Arctic but was on the edge of extinction in the 1970s due to the introduction of higher yield milk-producing breeds [28]. With current milk production relying almost exclusively on commercial Holstein or Ayrshire cattle in Finland, the less used native breeds face the risks of displacement and the loss of genetic diversity. A revival of the Northern Finncattle breed began in the 1980s, due to raised awareness of the alarming situation, and a gene bank herd was established for the Northern Finncattle in 1984. Over the past decade, there has been a growing interest in Northern Finncattle as a result of the breed’s regionally adapted characteristics. Efforts have been made to promote the farming of Northern Finncattle and the development of dairy products [29,30,31], alongside other native European breeds, making local dairy a more genetically divergent resource [32]. In addition to conservation schemes [29], it may be beneficial to investigate business opportunities based on the native breeds. 

To acquire in-depth knowledge about milk bioactive compounds, we characterized and compared the OS profiles and abundance of two commercial breeds, Holstein and Ayrshire (Nordic Red and Finnish Ayrshire), with the endangered Northern Finncattle breed. The findings gained are expected to help Finnish farmers diversify their cattle herds and may indicate opportunities for novel specialty milk production. Finally, it will raise awareness of the preservation effort to the dairy industry.

## 2. Materials and Methods

### 2.1. Milk Sample Collection

The milk samples for this study were obtained through an EU-funded project studying the milk and meat characteristics of the Northern Finncattle and the prevalent bovine breeds in Finland [33]. The aim was to find dairy farms with both Northern Finncattle and at least one other main breed in their herd. The dairy farms were selected based on the information collected. Healthy cows from 14 dairy farms in Finland with 19 Northern Finncattle (NF), 18 Ayrshire (AY), and 12 Holstein (HOL) were selected for this study. The lactation period, measured as days in milk (DIMs) ranged from 60 to 202 days (DIMs 116 ± 38, mean ± standard deviation). Table S1 provides the average values for the milk yield and the fat and protein contents of the selected samples. To ensure consistency, this study only included cows in their first to third lactations, with an average parity of 1.8 ± 0.7. The number of cows studied per farm varied between 2 and 5 individuals (see Table S2 for details). The criterion for sampling was a healthy cow, i.e., without any mastitis or other diseases. The milk samples were collected during the indoor feeding period of winter and spring from mid-December to early June. The morning milking was collected, and the samples were immediately cooled on the farms and transported frozen for storage in −80 °C freezers at Lapland Central Hospital in Rovaniemi. An overnight shipping from Finland to the United States was conducted in dry ice in an insulated box to ensure the samples remained frozen until reaching the University of California, Davis, for the OS extraction and analysis. In addition to analyzing all the individual samples, a pooled sample was prepared by mixing an equal volume of milk from all the cows in each breed to obtain a representative OS profile.

### 2.2. Oligosaccharide Extraction and Purification

Oligosaccharides were extracted and purified according to an established method [17]. The milk was mixed with an equal volume of nanopure water (Millipore, Burlington, MA, USA) and centrifuged at 13,000× *g* for 30 min at 4 °C. The skimmed milk was subjected to a Folch extraction using chloroform (Thermo Scientific, Waltham, MA, USA) /methanol (Alfa Aesar, Ward Hill, MA, USA) (2:1, *v*/*v*%) to remove any residual lipids/proteins. The mixture was further centrifugated, and the aqueous top layer containing the OS was carefully transferred to another tube, where cold ethanol was introduced to precipitate the proteins. The resulting supernatant was then transferred and evaporated using a vacuum concentrator at room temperature (Genevac SP Scientific, Warminster, PA, USA). The samples were reconstituted in nanopure water and purified by a solid-phase extraction using a HyperSep™ C18 microplate (ThermoFisher Scientific, Waltham, MA, USA). This step was necessary to remove the residual proteins and peptides. To desalt the samples, a Hypercarb™ porous graphitized carbon microplate (ThermoFisher Scientific) was employed. After activating the solid-phase material per the manufacturer’s instructions, the samples were loaded and then underwent a wash step with 4% acetonitrile (Fisher Scientific, Pittsburgh, PA, USA) (ACN) and 0.1% trifluoroacetic acid (Sigma-Aldrich, St. Louis, MO, USA) (TFA) in water (*v*/*v*/*v*) to eliminate the lactose and salts [34]. For the pooled samples of each breed, the OS were fractionated [35] by using sequential elution with 10% ACN–90% H_2_O (*v*/*v*), 20% ACN–80% H_2_O (*v*/*v*), and 40% ACN–0.1% TFA–59.9% H_2_O (*v*/*v*/*v*) and collecting each fraction separately to build a comprehensive library. A single step of elution with 40% ACN–0.1%TFA–59.9% H_2_O (*v*/*v*/*v*) was used for the individual samples to collect the overall OS for the relative quantification. The samples were analyzed at a 10-fold dilution to prevent mass spectrometry detector peak saturation. All the samples were spiked with an internal standard xylosyl–cellobiose at 1 mg/L concentration, purchased from Megazyme (Bray, Ireland).

### 2.3. Oligosaccharide Analysis by LC-QToF

The profiling analysis was performed with an Agilent 6520 accurate-mass Quadrupole-Time-of-Flight mass spectrometer, coupled to an Agilent 1200 liquid chromatography system featuring a microfluidic nano-electrospray chip interface (Agilent Technologies, Santa Clara, CA, USA). The chip used consisted of a 9 × 0.075 mm ID enrichment column and a 43 × 0.075 mm ID analytical column, both packed with 5 μm of porous graphitized carbon as the stationary phase. The mobile phase composition and elution gradient remained consistent for the MS and MS/MS methods. 

The chromatographic elution involved a binary gradient of (A) 3%ACN–0.1% formic acid in water (*v*/*v*/*v*) and (B) 90%ACN–0.1% formic acid in water (*v*/*v*/*v*). The column was equilibrated and eluted with a flow rate of 0.3 μL/min for the nano pump and 4 μL/min for the capillary pump. The 65 min gradient was programmed as follows: 0–2.5 min, 0% B; 2.5–20 min, 0–16% B; 20–30 min, 16–44% B; 30–35 min, 44–100% B; 35–45 min, 100% B; and 45–65 min, 0% B. 

The data acquisition occurred in the positive ionization mode with an *m/z* range of 450–2500. The electrospray capillary voltage was set at 1950V to maintain a steady spray. The pooled samples underwent MS/MS fragmentation to acquire the structural information. The individual samples were subject to MS analysis for an enhanced signal in the peak integration. The acquisition rate was 1 spectrum/s for both the MS and MS/MS. Automated precursor selection was employed based on abundance, with up to 8 precursors per MS. The precursor isolation window was at 4 *m/z*. The fragmentation energy was set at 1.3 V × *m/z* ÷ 100 with an offset of −3.5 V. Internal calibration was performed using *m/z* 922.0097 and 1221.9906 as the reference masses.

The software GlycoNote (https://github.com/MingqiLiu/GlycoNote, accessed on 26 September 2024) [36] was used to screen the MS/MS data and reveal the OS composition. Manual checks were performed on all the proposed identifications, and only the ones verified with the correct monosaccharide masses were included in the in-house library. 

### 2.4. Statistical Analysis

Each OS chromatographic peak was extracted, and the peak area (unitless) was integrated using MassHunter Profinder B10.0. The relative abundance was calculated by dividing the peak area of each OS by that of the internal standard. The differences in the OS contents between AY, HOL, and NF were analyzed with linear models using breed as the only explanatory factor on the SAS (version 9.4). The model residuals were assumed to be independent and normally distributed (log transformation for some OS) with constant variance. The Bonferroni method with α < 0.05 was used in the pairwise comparisons of the model-estimated breed means. The log-transformed OS means and their 95% confidence intervals were inversely transformed to the original scale for meaningful interpretation. The analyses were similar for the OS measured from only two breeds. The group mean and the box and whisker plots of different OS groups were made in GraphPad (version 10.2.0). The relative abundances for the individual fucosylated, neutral, and sialylated OS were compiled into three vectors containing the sum of relative abundances for each. These three vectors were scaled and utilized for 2D k-means clustering by Euclidean distances, as well as linear discriminant analysis (LDA). Both were performed with R software (version 4.2.3).

## 3. Results

### 3.1. Oligosaccharide Identification and Breed-Specific Library

**Figure 1:**
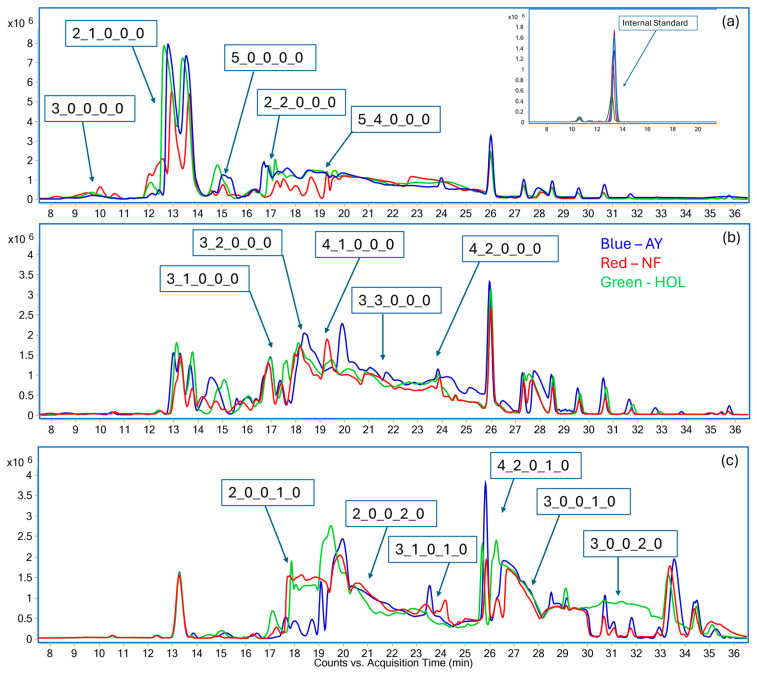
Overlay of base peak chromatograms (BPCs) for three different cow breeds subjected to fractionation. The BPCs are shown for (**a**) 10% ACN–90% H_2_O, (**b**) 20% ACN–80% H_2_O, and (**c**) 40% ACN–0.1% TFA–59.9% H_2_O fractions. Each chromatogram represents the comprehensive profiling of the oligosaccharides extracted from the respective breeds, highlighting differences in the oligosaccharide composition and concentration level across varying ACN fractions. The inset in (**a**) is the extracted peak of the internal standard. The OS compositions are described by their monosaccharide building blocks as a sequence of hexose (Glc/Gal)–N-acetylhexosamine (GlcNAc/GalNAc)–fucose–N-acetylneuraminic acid–N-glycolyneuraminic acid.

The OS profile of each breed, displayed as overlaid base peak chromatograms, is presented in Figure 1. This shows that more neutral oligosaccharides were eluted at the lower %ACN and all the acidic oligosaccharides were eluted with increasing the %ACN and adding TFA. The difference in signal intensity indicated the difference of OS abundance. The dominant peaks from each fraction collected using the solid-phase extraction also varied, meaning that fractionation resulted in unique elution profiles depending on the organic mobile phase composition. The first fraction, obtained using 10% ACN–90% H_2_O, showed the highest peak intensity (panel a) and contained only neutral OS, followed by the 20% ACN–80% H_2_O (panel b) and 40% ACN–0.1% TFA–59.9% H_2_O in water (panel c), with similar peak intensities and a more diverse profile, which included OS with NeuAc. This trend was consistent for all three breeds. The OS identified in each of the fractions for the same breed were complied to build a comprehensive breed-specific library, as shown in Table 1. The identification was not made based on the comparison with external runs of oligosaccharide standards, but by inspecting the actual fragmentation pattern of each oligosaccharide. As expected, the majority of OS belonged to the neutral (nonfucosylated and fucosylated) or sialylated classes, with only a few OS presenting the monosaccharide fucose. A total of 25 OS unique compositions, corresponding to up to 50 structures when considering isomers, were found in the three breeds, highlighting a high degree of similarity. None of the breeds contained OS with the monosaccharide NeuGc. The OS with ID 25 (composition 5_4_1_1_0), decorated with both fucose and NeuAc, was the largest and most complicated structure found in this dataset. The annotated structure of large fucosylated structures can be found in a previous study [37].

### 3.2. Relative Abundance

**Figure 2:**
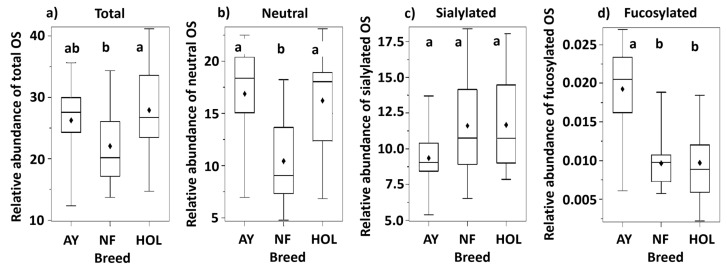
Box and whisker plots of the relative abundance for (**a**) total, (**b**) neutral, (**c**) sialylated, and (**d**) fucosylated oligosaccharides in the Ayrshire (AY), Northern Finncattle (NF), and Holstein (HOL) breeds. Different letters above the bars (a, b) indicate that the means of those groups are statistically significantly different, *p* < 0.05 (log transformation was used for the sialylated oligosaccharides).

**Figure 3:**
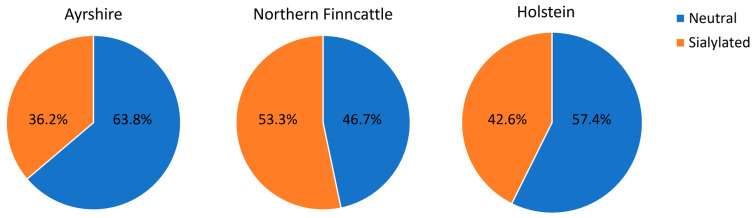
Pie charts displaying the average neutral and sialylated OS percentages in Ayrshire, Northern Finncattle, and Holstein.

The OS were grouped into several classes: neutral nonfucosylated, neutral fucosylated, fucosylated and sialylated, and sialylated nonfucosylated. A summary of the OS abundance for all classes is presented at the bottom of Table 2. This summary groups neutral fucosylated and sialylated fucosylated OS under the category ‘fucosylated’ and shows the relative abundance of each class as a proportion of the total, which is the sum of all the relative abundances. Therefore, the values were not reported in terms of the absolute amount for the sample. The pairwise comparisons between breeds are represented in Figure 2. The plots that do not share a common letter are significantly different with *p* < 0.05. The total OS abundances between breeds were not equal (*p* = 0.035). NF displayed lower overall abundance than HOL and AY. This was also true for NF neutral OS compared to both AY and HOL. A difference was indicated in the family-wise testing for the equality of means of the sialylated OS relative abundance between breeds (*p* = 0.042). The point estimates for HOL and NF (11.2) were higher than for AY (9.1). Considering that the majority of OS belong to the neutral or sialylated classes, it is important to note that NF had a higher percentage of sialylated than neutral OS from the calculation in Table 2. This is contrary to the two other breeds, as illustrated in Figure 3. In the neutral OS, composition 2_1_0_0_0 had the highest abundance among all three breeds. However, the abundance in AY and HOL is almost twice that in NF. This variation of 2_1_0_0_0 has been reported in other native cattle breeds [27]. AY had three fucosylated structures, making it the breed with the highest number of fucosylated species, although, as expected for bovine milk, fucosylated OS were substantially lower than neutral nonfucosylated and sialylated. OS 5_4_1_1_0 was not included in the following comparison since it was only found in one breed.

### 3.3. Clustering and Categorization Methods

**Figure 4:**
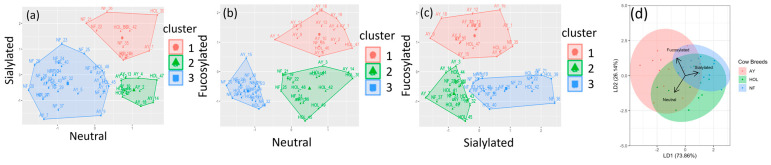
Two-dimensional k-means clustering for fucosylated, sialylated, and neutral oligosaccharide relative abundance in Ayrshire (AY), Holstein (HOL), and Northern Finncattle (NF) breeds. (**a**) Clusters generated from the relative abundance of sialylated vs. neutral oligosaccharide. (**b**) Clusters generated from the relative abundance of fucosylated vs. sialylated oligosaccharide. (**c**) Clusters generated from the relative abundance of fucosylated vs. neutral oligosaccharide. (**d**) Linear discriminant analysis using the relative abundance of fucosylated, sialylated, and neutral oligosaccharide classes.

The relative abundances for the individual neutral nonfucosylated, neutral fucosylated, and sialylated OS were compiled into three vectors containing the total relative abundance for each breed. These three vectors were scaled and utilized for the two-dimensional k-means clustering in Figure 4a–c, and the proportions of each breed in the clusters are presented in Table 3. The shaded clusters in the k-means models represent groups determined by the Euclidean distances between the OS coordinates of all the cows in this study. The proportions of NF in the clusters of neutral vs. sialylated, neutral vs. fucosylated, and fucosylated vs. sialylated were 0.79, 0.63, and 0.63, respectively. The proportions of AY in the clusters of neutral vs. fucosylated and fucosylated vs. sialylated were 0.72 and 0.78, respectively, whereas the highest proportion of HOL samples was only 0.58 in neutral vs. fucosylated. The clustering results indicate that although HOL OS may be less distinct from AY or NF, they may be more encompassing of different types. 

The LDA model was based on the sialylated, fucosylated, and neutral OS relative abundances in the three breeds and was trained on a 75% subset of samples (n = 49) and tested on the remaining 25%, yielding a prediction accuracy of 0.7. Leave-one-out cross-validation was run on the entire dataset, yielding a prediction accuracy of 0.7143 with a 95% confidence interval of (0.5674, 0.8342). This indicates that the breed may be predicted with an accuracy of approximately 71.4%, suggesting distinguishable differences in the milk OS content of AY, NF, and HOL. The colored eclipses represent the breeds. The overlap of the HOL ellipse with that of the AY and NF in Figure 4d indicates similarities in the HOL milk OS content to that of both the AY and NF milk, making it more difficult to categorize.

## 4. Discussion

Holsteins are currently the most globally distributed breed and they are commonly used in Finland. Over time, they have been bred to produce the highest volume of milk. Ayrshires also play a significant role in the Finnish dairy industry, being the second most common breed. They are highly valued for their high milk yield and milk quality with a good balance of fat and protein, as well as good fertility [38]. The number of reproducing stocks of Northern Finncattles is slowly rising due to a national preservation program. In addition to high-quality milk, farmers value Northern Finncattles for their higher fertility, lower infection rates, and overall longevity, as well as their excellent adaptation to grazing [39]. Studying specific features of native breeds is increasingly important for cattle breeding in response to climate change challenges, species extinction, and biodiversity loss. There is a wealth of tradition and practitioner’s knowledge on native breeds that can be leveraged and combined with research to promote their revival and generate new income opportunities for farmers [40]. Milk composition is undoubtedly an important aspect of understanding the breed characteristics. The synthesis of milk OS is a phenotypic trait that cannot be solely explained by genetics. However, few studies have mapped out the causal variants, genomic regions, and candidate genes associated with major OS production [41,42]. Advanced analytical technologies have once again proven useful in deciphering the milk OS composition and abundance. 

This study aligns with previous works on milk OS comparison focused on breeds. Our group previously reported that Jersey cattle produced substantially more diverse OS than Holstein [26]. Western and Eastern Finncattle milk OS have also been characterized and showed inter-breed variations [27]. This study contributed new knowledge on the types and abundance of OS in Ayrshire (AY), Holstein (HOL), and Northern Finncattle (NF) breeds.

The clustering and categorization analyses described in this study (Figure 4) indicate that AY and NF possess an OS profile and abundance distinct from Holstein. The higher abundance in neutral and fucosylated OS structures in AY makes it stand out, and it may be linked to the activity of a fucose transferase gene [43]. Kantanen et al. [42] studied the genetic diversity of many breeds and found that North European breeds were significantly different and that their gene pools were developed through a breed-specific evolution. In that work, AY and HOL fell in the same cluster, while NF fell into the Northern indigenous breed group. That could potentially explain the similarity in total OS abundance in milk from AY and HOL observed in this present work, whereas NF was statistically lower. The higher sialylated OS proportion seen in NF is a desirable feature if a larger overall amount of oligosaccharides was to be produced. While many animal studies associated sialylated OS with improved brain development [44,45], one short-term study did not find evidence for OS incorporation into the brain [46]. Other studies have shown that sialylated OS help establish a healthy gut microbiome [47,48]. The sialic acid bound to OS could also be a source of building blocks for the synthesis of other glycoconjugates related to brain health [44,49] (such as recognition and memory abilities). In addition, the higher relative abundance of sialylated OS in NF milk and the lower abundance of fucosylated in NF compared to AY potentially indicates different synthetic pathways that warrant additional investigation.

It is also important to acknowledge other known factors that influence OS levels, for example, cow feed [23,24]. Feed was not strictly controlled in this study because the samples came from several farms. All the samples in this study were collected during the indoor feeding season and thus none of the animals grazed on pasture. Additionally, samples were taken from at least two breeds at each farm with the exception of one farm where samples were taken from only two HOL individuals (see Table S2). Lactation stage is another OS-influencing factor that has been accounted for in this study. Early lactation milk contains a greater level of OS compared to mature milk [22,50]. Our study design excluded the variability of the seasonality factor since the samples were acquired from multiple farms during the indoor feeding period of winter and spring. The results reflected the average of the OS level throughout this milking season. The effect of grazing during the summer season would warrant a further study as it has been shown that the total sialic acid concentration increased as a result of grazing management [23].

It is also worth acknowledging that fewer HOL samples were analyzed in this study. This decision was made because there is already a wealth of research that has been performed on this globally distributed breed [26,51,52]. Thus, we decided to allocate time and resources towards less-studied breeds. A greater number of samples would likely improve the 2D k-means and LDA models. Lastly, performing an absolute quantification of OS with the few available standards and comparing with milk samples from other countries would provide a more comprehensive analysis.

## 5. Conclusions

This study compared milk oligosaccharide profiles of the commercial breeds Ayrshire, Holstein, and native Northern Finncattle. Milk OS from these breeds varied in the abundance of neutral, sialylated, and fucosylated categories. Two-dimensional k-means clustering showed Ayrshire and Northern Finncattle in somewhat distinct clusters, whereas Holstein overlapped more with the other breeds. These findings add new knowledge to previous reports on breed-dependent milk oligosaccharide composition. The observed differences may be partially explained by the genetic makeup of these breeds.

The oligosaccharide profiles identified in this study have potential implications for breeding Ayrshire to obtain more diverse oligosaccharide structures to boost the diversity of gut microbiome, as well as for breeding Northern Finncattle for dairy with a higher proportion of sialylated structures, which may improve consumers’ brain and gut health. Further research is needed to explore other less studied cattle breeds to expand the field of milk composition analysis and the nutritional values of its components. This points towards opportunities in specialty dairy products with bonus health attributes and the dairy industry at large.

## Figures and Tables

**Table 1:** Oligosaccharide library for Northern Finncattle, Ayrshire, and Holstein obtained by analyzing a pooled sample for each breed by LC-MS, detailing the monomeric composition, monoisotopic mass, and number of isomers identified in each breed.

OS ID	Composition	Monoisotopic Mass	Northern Finncattle	Ayrshire	Holstein
			Number of Isomers		
1	3_0_0_0_0	504.1690	5	5	5
2	2_1_0_0_0	545.1956	4	4	4
3	2_0_0_1_0	633.2116	2	2	2
4	4_0_0_0_0	666.2219	4	4	4
5	1_1_0_1_0	674.2382	1	1	1
6	3_1_0_0_0	707.2484	4	4	4
7	2_2_0_0_0	748.2750	3	3	3
8	3_0_0_1_0	795.2645	2	2	2
9	5_0_0_0_0	828.2747	3	3	3
10	2_1_0_1_0	836.2910	1	1	1
11	4_1_0_0_0	869.3012	3	2	2
12	3_2_0_0_0	910.3278	2	2	2
13	2_0_0_2_0	924.3070	1	1	1
14	6_0_0_0_0	990.3275	3	3	3
15	3_1_0_1_0	998.3438	1	1	1
16	4_2_0_0_0	1072.3806	2	2	2
17	3_0_0_2_0	1086.3599	1	1	1
18	3_3_0_0_0	1113.4072	1	1	1
19	2_1_0_2_0	1127.3864	1	1	1
20	4_1_0_1_0	1160.3967	1	1	1
21	4_2_0_1_0	1363.4760	1	1	1
22	5_4_0_0_0	1640.5922	1	1	1
23	5_4_1_0_0	1786.6501	1	1	1
24	3_6_1_0_0	1868.7032	1	1	1
25	5_4_1_1_0	2077.7455	1	1	1
**Number of isomers**			50	49	49

**Table 2:** Relative abundance of oligosaccharides in the milk of Ayrshire, Northern Finncattle, and Holstein.

	AY ^1^	95% CI ^2^	NF^1^	95% CI ^2^	HOL ^1^	95% CI ^2^	*p*-Value
**Neutral oligosaccharides**							
**3_0_0_0_0**	2.9 ^a^	2.6, 3.1	2.4 ^b^	2.2, 2.6	2.8 ^ab^	2.5, 3.1	0.019
**2_1_0_0_0**	9.0 ^a^	7.2, 10.9	4.4 ^b^	2.6, 6.2	9.5 ^a^	7.2, 11.7	<0.001
**4_0_0_0_0**	3.5	2.8, 4.3	2.4	1.7, 3.1	2.8	1.9, 3.7	0.080
**3_1_0_0_0**	0.44 ^a^	0.39, 0.49	0.35 ^b^	0.30, 0.40	0.36 ^ab^	0.30, 0.43	0.028
**2_2_0_0_0**	0.087	0.073, 0.101	0.079	0.066, 0.093	0.100	0.083, 0.117	0.173
**5_0_0_0_0 ^3^**	0.024	0.018, 0.027	0.020	0.015, 0.022	0.021	0.015, 0.025	0.312
**4_1_0_0_0 ^3^**	0.36 ^ab^	0.21, 0.37	0.34 ^a^	0.24, 0.41	0.21 ^b^	0.13, 0.26	0.047
**3_2_0_0_0 ^3^**	0.054 ^a^	0.033, 0.058	0.048 ^a^	0.031, 0.054	0.020 ^b^	0.012, 0.024	<0.001
**6_0_0_0_0**	0.35 ^a^	0.30, 0.39	0.25 ^b^	0.20, 0.29	0.34 ^ab^	0.28, 0.39	0.008
**4_2_0_0_0**	0.050	0.040, 0.060	0.037	0.028, 0.047	0.034	0.022, 0.046	0.094
**3_3_0_0_0 ^3^**	0.032	0.023, 0.043	0.025	0.019, 0.034	0.022	0.016, 0.032	0.322
**5_4_0_0_0**	0.008	0.006, 0.010			0.009	0.007, 0.012	0.409
**Neutral fucosylated** **oligosaccharides**							
**5_4_1_0_0**	0.012	0.010, 0.014	0.010	0.008, 0.011	0.010	0.007, 0.012	0.183
**3_6_1_0_0**	0.007	0.006, 0.009					
**Fucosylated and sialylated oligosaccharides**							
**5_4_1_1_0**	0.005	0.005, 0.006					
**Sialylated oligosaccharides**							
**2_0_0_1_0 (α-2,6 isomer)**	1.6	1.2, 2.0	2.0	1.6, 2.4	2.0	1.5, 2.5	0.195
**2_0_0_1_0 (α-2,3 isomer)**	4.3	3.8, 4.9	4.8	4.3, 5.3	4.5	3.8, 5.2	0.477
**1_1_0_1_0 ^3^**	0.074	0.045, 0.087	0.112	0.057, 0.108	0.108	0.06, 0.134	0.348
**3_0_0_1_0**	0.64	0.56, 0.72	0.52	0.44, 0.60	0.58	0.48, 0.68	0.109
**2_1_0_1_0 ^3^**	0.032 ^b^	0.023, 0.039	0.014 ^c^	0.009, 0.016	0.092 ^a^	0.045, 0.089	<0.001
**2_0_0_2_0**	2.2 ^b^	1.8, 2.5	3.6 ^a^	3.3, 4.0	3.5 ^a^	3.0, 3.9	<0.001
**3_1_0_1_0**	0.021	0.016, 0.026			0.025	0.019, 0.031	0.238
**3_0_0_2_0 ^3^**	0.425 ^b^	0.236, 0.428	0.396 ^ab^	0.266, 0.473	0.656 ^a^	0.405, 0.837	0.035
**4_1_0_1_0**	0.073	0.053, 0.092	0.081	0.062, 0.100	0.047	0.023, 0.071	0.080
**4_2_0_1_0 ^3^**	0.015	0.012, 0.021	0.022	0.017, 0.030	0.017	0.012, 0,024	0.165
**2_1_0_2_0**					0.13	0.08, 0.18	
**Total oligosaccharides**	26.2 ^ab^	23.2, 29.3	22.0 ^b^	19.1, 25.0	27.9 ^a^	24.2, 31.6	0.035
**Neutral oligosaccharides**	16.9 ^a^	14.8, 19.0	10.4 ^b^	8.4, 12.5	16.2 ^a^	13.6, 18.8	<0.001
**Sialylated oligosaccharides ^3^**	9.1	8.0, 10.3	11.2	9.9, 12.7	11.2	9.6, 13.1	0.042
**Fucosylated oligosaccharides**	0.019 ^a^	0.017, 0.022	0.010 ^b^	0.007, 0.012	0.010 ^b^	0.007, 0.012	<0.001
**Neutral/Total oligosaccharides**	63.6 ^a^	59.8, 67.4	46.7 ^b^	43.0, 50.4	57.3 ^a^	52.6, 61.9	<0.001
**Sialylated/Total oligosaccharides**	36.3 ^b^	32.5, 40.1	53.3 ^a^	49.6, 57.0	42.7 ^b^	38.0, 47.3	<0.001
**Fucosylated/Total oligosaccharides**	0.074 ^a^	0.066, 0.082	0.046 ^b^	0.038, 0.054	0.035 ^b^	0.025, 0.045	<0.001

^1^ Mean estimates within a row without a common letter (a, b, and c) differ, *p* < 0.05. ^2^ 95% confidence interval. ^3^ Log transformation was used, and the estimated means and CIs for the means were transformed back to the original scale.

**Table 3:** Proportion of breeds in each cluster.

Neutral vs. Sialylated	AY	NF	HOL	Neutral vs. Fucosylated	AY	NF	HOL	Sialylated vs. Fucosylated	AY	NF	HOL
Cluster	n = 18	n = 19	n = 12	Cluster	n = 18	n = 19	n = 12	Cluster	n = 18	n = 19	n = 12
1	0.17	0.21	0.33	1	0.72	0.05	0.17	1	0.78	0.05	0.17
2	0.44	0	0.25	2	0.17	0.32	0.58	2	0.22	0.32	0.42
3	0.39	0.79	0.42	3	0.11	0.63	0.25	3	0	0.63	0.42

## Data Availability

The supporting data are available on request from the corresponding author.

## Supplementary Materials

The following supporting information can be downloaded at https://www.mdpi.com/article/10.3390/biology13110855/s1, Table S1: average values for milk yield and fat and protein content in milk of Ayrshire, Northern Finncattle, and Holstein; and Table S2: information on the basic composition of milk and the characteristics of the cows selected for this study.
