# Supplementary material for: A Comparative Analysis of Milk Oligosaccharides via LC-MS: Globally Distributed Cattle Breeds and Native Northern Finncattle
Source: Biology (Basel). 2024 Oct 23;13(11):855. doi: 10.3390/biology13110855 (PMC11592061; doi:10.3390/biology13110855)
Supplement: Supplementary file 1 [file biology-13-00855-s001.zip › biology-3198276-supplementary.pdf]

Table S1. Average values for milk yield and fat and protein content in milk of Ayrshire, Northern Finncattle, and Holstein.

	AY[1]	95 % CI[2]	NF[1]	95 % CI[2]	HOL[1]	95 % CI[2]	*p*-value
Milk yield, kg/d	30.2[a]	26.3, 34.0	18.4[b]	14.7, 22.2	34.0[a]	29.2, 38.7	<0.001
Fat, g/100 g	4.78	4.26, 5.30	4.31	3.80, 4.82	4.09	3.45, 4.73	0.217
Protein, g/100 g	3.47	3.33, 3.61	3.42	3.28, 3.55	3.26	3.10, 3.43	0.151

[1]Mean estimates within a row without a common letter (a, b) differ, $p < 0.05$.
[2]95 % confidence interval

Table S2. Information on the basic composition of milk and the characteristics of the cows selected for this study.

Sample Name	Somatic Cell Counts	Fat %	Protein %	Farm	DIM	Sampling date	Parity	Milk Yield (daily)
AY_1	116.00	4.34	3.28	1.00	127.00	2/4/2021	2.00	36.40
AY_2	44.00	4.30	3.07	1.00	163.00	2/4/2021	2.00	30.60
AY_3	37.00	8.03	3.75	3.00	176.00	6/15/2021	1.00	30.60
AY_4	8.00	3.95	3.46	4.00	108.00	4/13/2021	1.00	31.30
AY_5	24.00	4.08	3.27	5.00	78.00	3/26/2021	1.00	30.40
AY_6	19.00	4.36	3.53	5.00	60.00	3/26/2021	3.00	40.90
AY_7	15.00	8.63	3.30	6.00	169.00	5/26/2021	1.00	23.40
AY_8	32.00	3.80	3.65	7.00	79.00	5/8/2021	2.00	43.00
AY_9	30.00	3.98	2.89	8.00	64.00	6/5/2021	1.00	28.80
AY_10	18.00	4.04	3.54	10.00	72.00	5/26/2021	1.00	23.40
AY_11	47.00	4.93	3.86	10.00	124.00	5/26/2021	2.00	26.40
AY_12	-	5.08	3.67	11.00	79.00	12/15/2021	1.00	31.20
AY_13	-	5.95	3.99	11.00	145.00	12/15/2021	1.00	27.60
AY_14	17.99	3.42	2.89	12.00	93.00	1/1/2022	3.00	35.40
AY_15	-	5.20	3.90	15.00	143.00	3/24/2022	2.00	30.00
AY_16	-	4.70	3.50	15.00	192.00	3/24/2022	2.00	40.30
AY_17	-	3.25	3.33	19.00	119.00	6/14/2022	2.00	23.00
AY_18	-	3.97	3.58	19.00	98.00	6/14/2022	3.00	10.00
NF_1	176.00	3.76	3.06	1.00	123.00	2/4/2021	2.00	25.00
NF_2	17.00	4.24	3.26	2.00	72.00	3/27/2021	1.00	16.00
NF_3	11.00	3.85	3.35	2.00	118.00	3/27/2021	2.00	16.00
NF_4	53.00	5.05	3.46	2.00	131.00	3/27/2021	2.00	11.40
NF_5	233.00	4.77	3.48	2.00	191.00	3/27/2021	2.00	7.40
NF_6	33.00	3.46	3.10	2.00	65.00	3/27/2021	3.00	20.30
NF_7	45.00	3.32	3.08	3.00	69.00	6/15/2021	3.00	31.20
NF_8	200.00	4.21	3.57	4.00	116.00	4/13/2021	1.00	15.50
NF_9	43.00	4.34	3.44	4.00	121.00	4/13/2021	1.00	21.90

NF_10	19.00	3.92	3.20	5.00	103.00	3/26/2021	1.00	20.30
NF_11	18.00	4.48	3.57	5.00	92.00	3/26/2021	3.00	22.30
NF_12	13.00	4.40	3.70	6.00	98.00	5/26/2021	2.00	14.90
NF_13	12.00	4.81	3.98	7.00	202.00	5/8/2021	1.00	15.00
NF_14	17.00	4.02	3.13	7.00	107.00	5/8/2021	3.00	24.80
NF_15	26.00	5.35	3.59	8.00	159.00	6/5/2021	1.00	13.80
NF_16	29.00	5.24	3.32	10.00	99.00	5/26/2021	1.00	18.20
NF_17	-	5.26	3.81	11.00	152.00	12/15/2021	1.00	11.80
NF_18	-	4.20	3.70	15.00	81.00	3/24/2022	2.00	35.20
NF_19	-	3.18	3.13	19.00	106.00	6/14/2022	2.00	9.00
HOL_1	76.00	3.08	3.06	1.00	98.00	2/4/2021	2.00	33.60
HOL_2	35.00	4.73	3.65	1.00	186.00	2/4/2021	2.00	32.40
HOL_3	20.00	5.68	3.42	2.00	72.00	3/27/2021	1.00	29.40
HOL_4	16.00	3.97	3.36	4.00	150.00	4/13/2021	1.00	34.10
HOL_5	6.00	3.02	2.93	4.00	166.00	4/13/2021	1.00	41.80
HOL_6	22.00	3.99	3.57	5.00	136.00	3/26/2021	1.00	38.50
HOL_7	-	4.46	3.14	11.00	84.00	12/15/2021	2.00	51.00
HOL_8	17.48	3.64	3.03	12.00	91.00	1/1/2022	2.00	19.00
HOL_9	6.00	3.84	3.45	14.00	112.00	3/16/2022	2.00	33.98
HOL_10	25.00	6.31	2.87	14.00	86.00	3/16/2022	3.00	48.68
HOL_11	-	3.36	3.17	19.00	84.00	6/14/2022	2.00	25.00
HOL_12	-	3.04	3.49	19.00	134.00	6/14/2022	2.00	20.00
